# Assessing healthcare providers' knowledge and practices relating to insecticide-treated nets and the prevention of malaria in Ghana, Laos, Senegal and Tanzania

**DOI:** 10.1186/1475-2875-10-363

**Published:** 2011-12-13

**Authors:** Steven J Hoffman, G Emmanuel Guindon, John N Lavis, Godwin D Ndossi, Eric JA Osei, Mintou Fall Sidibe, Boungnong Boupha

**Affiliations:** 1 McMaster Health Forum, McMaster University, Hamilton, Ontario, Canada; 2 Department of Clinical Epidemiology and Biostatistics, McMaster University, Hamilton, Ontario, Canada; 3 Global Health Diplomacy Program, Munk School of Global Affairs, University of Toronto, Toronto, Ontario, Canada; 4 Interfaculty Initiative in Health Policy, Harvard University, Cambridge, MA, USA; 5 Propel Centre for Population Health Impact, University of Waterloo, Waterloo, Ontario, Canada; 6 Centre for Health Economics and Policy Analysis, McMaster University, Hamilton, Ontario, Canada; 7 Department of Political Science, McMaster University, Hamilton, Ontario, Canada; 8 Tanzania Food and Nutrition Centre, Dar es Salaam, Tanzania; 9 Council for Scientific and Industrial Research Secretariat, Accra, Ghana; 10 Direction des Études de la Recherche et de la Formation, Comité National d' Éthique, Dakar, Senegal; 11 National Institute of Public Health, Ministry of Health, Vientiane, Lao People's Democratic Republic

## Abstract

**Background:**

Research evidence is not always being disseminated to healthcare providers who need it to inform their clinical practice. This can result in the provision of ineffective services and an inefficient use of resources, the implications of which might be felt particularly acutely in low- and middle-income countries. Malaria prevention is a particularly compelling domain to study evidence/practice gaps given the proven efficacy, cost-effectiveness and disappointing utilization of insecticide-treated nets (ITNs).

**Methods:**

This study compares what is known about ITNs to the related knowledge and practices of healthcare providers in four low- and middle-income countries. A new questionnaire was developed, pilot tested, translated and administered to 497 healthcare providers in Ghana (140), Laos (136), Senegal (100) and Tanzania (121). Ten questions tested participants' knowledge and clinical practice related to malaria prevention. Additional questions addressed their individual characteristics, working context and research-related activities. Ordinal logistic regressions with knowledge and practices as the dependent variable were conducted in addition to descriptive statistics.

**Results:**

The survey achieved a 75% response rate (372/497) across Ghana (107/140), Laos (136/136), Senegal (51/100) and Tanzania (78/121). Few participating healthcare providers correctly answered all five knowledge questions about ITNs (13%) or self-reported performing all five clinical practices according to established evidence (2%). Statistically significant factors associated with higher knowledge within each country included: 1) training in acquiring systematic reviews through the Cochrane Library (OR 2.48, 95% CI 1.30-4.73); and 2) ability to read and write English well or very well (OR 1.69, 95% CI 1.05-2.70). Statistically significant factors associated with better clinical practices within each country include: 1) reading scientific journals from their own country (OR 1.67, 95% CI 1.10-2.54); 2) working with researchers to improve their clinical practice or quality of working life (OR 1.44, 95% CI 1.04-1.98); 3) training on malaria prevention since their last degree (OR 1.68, 95% CI 1.17-2.39); and 4) easy access to the internet (OR 1.52, 95% CI 1.08-2.14).

**Conclusions:**

Improving healthcare providers' knowledge and practices is an untapped opportunity for expanding ITN utilization and preventing malaria. This study points to several strategies that may help bridge the gap between what is known from research evidence and the knowledge and practices of healthcare providers. Training on acquiring systematic reviews and facilitating internet access may be particularly helpful.

## Background

There is growing awareness and concern among educators, researchers, practitioners and policymakers that what is known from research evidence is often not being put into action [1,2]. An expanding number of studies continue to show that research evidence is not being disseminated to healthcare providers who need it to inform their clinical practice and improve the health of their patients. Not only does this knowledge deficit lead to sub-optimal care, but it can result in the provision of ineffective services, inefficient use of resources and increasing inequities in health outcomes. This reality is particularly devastating for low-and middle-income countries which suffer from greater resource limitations than more affluent high-income countries. This situation is particularly salient when there are several cost-effective interventions that exist to prevent and address some of today's greatest global health challenges [3]. They are just not all being appropriately utilized.

Efforts to address malaria are particularly implicated by this "know-do" gap given the proven effectiveness of insecticide-treated nets (ITNs) in preventing the disease [4-6], this intervention's cost-effectiveness [7-10], and disappointing patterns in their utilization. The World Malaria Report 2009 highlights that only 31% of African households own at least one ITN and that only 24% of children (< 5 years) used an ITN for at least one day in 2008 [11]. These statistics are well below the World Health Assembly's target of 80% coverage [12]. Research shows that intensive malaria control, and particularly preventative ITNs, can help countries meet the Millennium Development Goals of reducing child mortality by two-thirds (Goal 4) and reversing malaria's incidence worldwide (Goal 6) [3,12-14]. The full implementation of existing malaria interventions like ITNs is also expected to contribute to eradicating extreme poverty and hunger (Goal 1), achieving universal primary education (Goal 2), improving maternal health (Goal 5), and developing a global partnership for development (Goal 8), which includes access to affordable drugs [7,15]. Immediate action is clearly necessary.

This study seeks to probe the gap between what is known globally through research evidence about malaria prevention interventions (specifically ITNs) and the related knowledge and practices of healthcare providers in low- and middle-income countries. While many studies have asked *community members *about their knowledge and practices relating to malaria prevention [16-39], none could be found that asked these same questions of healthcare providers--who are intuitively and empirically known to influence their patients' behaviour in a variety of contexts [40-44]. In contrast, many studies of healthcare providers' knowledge and practices have addressed malaria diagnosis, treatment, management and transmission [45-50], and the prevention of other conditions, including cancer [51-60], dehydration from diarrhea [61-68] and pregnancy [69-73]. A better understanding of the factors that influence whether healthcare providers in low- and middle-income countries are knowledgeable about and optimally practice malaria prevention will serve to inform efforts aiming to eliminate the global burden of this disease for the future.

## Methods

This inquiry is part of a larger study sponsored by the World Health Organization that examined the link between research, practice and policy. Other elements of this global effort have focused on whether and how healthcare providers use research evidence [74,75] and the extent to which researchers support its use [76,77]. This is the first time that the collected data on healthcare providers' knowledge and practices has been analysed and presented.

### Questionnaire design

The development, translation, pilot-testing, reliability and validity of the questionnaire administered in this study has been described elsewhere [74]. In summary, the instrument was based on nine existing questionnaires [78-86]. The survey instrument included items about the respondents' individual characteristics, working context, training, networking activities, and access to, trust in and use of research evidence. There were also five true/false questions testing respondents' knowledge of malaria prevention and five questions assessing relevant clinical practices that were developed from multiple sources [87-90]. The instrument was found to have high internal consistency (i.e., reliability) and content and face validity. It was translated into Lao and French for administration in Laos and Senegal [74].

### Data collection

The questionnaire was administered locally between October 2004 and December 2005 by country teams in Ghana, Laos, Senegal and Tanzania with each aiming to obtain complete responses from 100 healthcare providers. These four low- and lower-middle-income countries differ in population size, per capita income, health expenditures, life expectancy, coverage for ITNs, and computer and internet access (Table 1).

**Table 1:** Country Profiles in 2005

Country	Ghana	Laos	Senegal	Tanzania	Source
Population (in millions)	22	6	12	38	[91]

GDP per capita (in PPP int'l $)	2, 607	2, 147	1, 926	750	[92]

Per capita total expenditure on health (in PPP int'l $)	95	74	72	29	[93]

Per capita government expenditure on health (in PPP int'l $)	40	15	29	12	[93]

Life expectancy at birth for males/females (in years)	58/59	61/63	60/64	49/51	[94]

Children under-five mortality rate (per 1, 000 live births)	112	79	136	122	[94]

Children under five sleeping under a net (%, 2003-2006)	33	82	14	31	[95]

Children under five sleeping under an ITN (%, 2003-2006)	22	18	7	16	[95]

Personal computers per population (%, 2004)	1	0	2	1	[94]

Internet users per population (%, 2004)	2	0	5	1	[94]

Data are for 2005 unless otherwise indicated

In Ghana, the country team constructed a sampling frame of appropriate healthcare providers from lists of government and private health facilities obtained from the Ministry of Health and the Society of Private Medical & Dental Practitioners respectively. A stratified cluster random sampling process was used with the facilities stratified by region (Greater Accra and Ashanti) and governance (public *vs*. private) and then selected randomly for inclusion. 140 healthcare providers were approached representing 48 different facilities. In Laos, a sampling frame was constructed based on existing lists of providers from the Ministry of Health and four provincial health departments. A stratified random sampling process was similarly applied to select 136 healthcare providers who worked at different types of facilities (central, provincial and district hospitals) in Borikhamsay, Savannakheth and Vientiane. In Senegal, a sampling frame was developed from lists of providers working at eight Dakar- or Thiès-based institutions and a list of clinician-scientists they had previously constructed. Simple random sampling was used to select 100 healthcare providers. Finally, in Tanzania, country investigators used purposive sampling to identify 121 regional or district medical officers who were attending a meeting for malaria and the integrated management of childhood illness. These variations in sampling frames and methodology, and the limited sample size, preclude meaningful between-country comparisons.

### Data analysis

Basic descriptive statistics were calculated for relevant items. In addition, simple ordinal logistic models were used to explore factors associated with the healthcare providers' knowledge and practices relating to ITNs and malaria prevention. Composite knowledge scores were constructed for each respondent based on the proportion of the five true/false knowledge-testing questions that they answered correctly. All questions were weighted equally and no penalty was given for incorrect answers. Similarly, composite practice scores were constructed for each respondent based on the frequency in which they reported doing the five practices on a five-point scale (i.e., 1 "never", 2 "rarely", 3 "sometimes", 4 "often", and 5 "very often"). The scale was inverted for the one practice which is contrary to recommended practice. All questions were weighted equally such that individual practice scores were integers ranging from five to 25. Both knowledge and practice scores were transformed into quintiles (within each country) and used as ordinal variables. Independent variables for both models included healthcare providers' 1) use of particular sources of evidence, 2) views and activities related to improving clinical practice, and 3) individual and practice characteristics. Multiple imputation (using 100 imputations) was used to fill in missing values using multivariate normal regressions. This approach was selected because it accommodates arbitrary missing-value patterns [96]. Observations were excluded when the dependent variable was missing. All statistical analyses were conducted using Stata/MP 11.2 for Macintosh.

In the analysis, systematic reviews (and the Cochrane Library as the single most comprehensive source) are emphasized because they are widely recognized as the best available approach to the synthesis of global research evidence, they efficiently provide summary information to inform decision-making, and they are widely-available and internationally authoritative on clinical interventions [97-99], including ITNs for the prevention of malaria [4-6]. Previous studies informed the decision to differentiate between scientific journals from high-income countries and the respondents' own country as well as between full reports and summaries [75,80,100].

## Results

372 healthcare providers participated in this study out of the 497 providers approached, yielding an overall response rate of 75% (107 of 140 from Ghana, 136 of 136 from Laos, 51 of 100 from Senegal, and 78 of 121 from Tanzania).

A majority of the respondents were male (56%), trained as primary care physicians (67%), could read and write English (63%), and worked in rural areas (56%), government-operated facilities (86%) and hospitals (82%). On average, the respondents were 42 years old and spent the greatest percentage of their time on clinical practice (59%) rather than research (8%), teaching (10%) or administrative duties (18%). Few had easy access to a computer with a CD ROM (24%) or the internet (22%), had earned master's or doctorate degrees (15%), and worked with researchers (44%). Few of the respondents had training on acquiring, assessing or adapting research evidence since their last degree although a substantial number received training related specifically to malaria prevention (45%). There were also few respondents that self-reported using the electronic Cochrane Library at least once a month (11%), but relatively more that self-reported reading electronic or paper versions of clinical practice guidelines, protocols or decision-support tools (59%), scientific journals from either their own country (55%) or high-income countries (36%), and summaries of articles, reports and reviews from public or non-profit organizations (57%). Almost half of the participating healthcare providers reported that research performed in their own country was of above average or excellent quality (48%) (Table 2).

**Table 2:** Descriptive statistics on healthcare providers' individual characteristics, working context, and views about and use of research evidence

Factor	All N = 372	Ghana N = 107	Laos N = 136	Senegal N = 51	Tanzania N = 75
**Individual characteristics**					

Age, yr, mean	42.0	43.0	40.7	38.9	45.5

Sex, male	55.9	59.8	41.2	45.1	84.9

Type of health care provider					

General practitioner	67.2%	53.8%	86.6%	29.4%	77.8%

Specialist physician	5.8%	10.4%	4.5%	5.9%	1.4%

Nurse	15.7%	31.1%	2.2%	37.3%	2.8%

Health worker	6.6%	2.8%	5.2%	17.6%	6.9%

Other	4.7%	1.9%	1.5%	9.8%	11.1%

**Allocation of time, % of time†**					

Clinical practice	59.0%	71.0%	65.0%	63.6%	28.4%

Research	7.7%	7.6%	6.4%	15.8%	4.8%

Teaching	10.3%	10.2%	9.5%	6.6%	14.4%

Administration	18.2%	9.2%	12.8%	8.5%	47.2%

Master's or doctorate degree	15.1%	15.0%	8.1%	7.8%	32.1%

Training (since completed last degree) in:					

Acquiring systematic reviews through the Cochrane Library	8.3%	12.1%	4.4%	4.1%	19.4%

Critically appraising systematic reviews	10.8%	16.8%	3.7%	6.3%	33.3%

Integrated Management of Childhood Illness (IMCI)	34.9%	43.3%	14.0%	39.2%	72.9%

Prevention of malaria	44.6%	39.2%	30.9%	74.5%	65.1%

Easy access to personal computer with CD ROM	24.3%	22.6%	7.5%	31.4%	56.3%

Easy access to Internet	21.7%	21.8%	6.7%	43.1%	35.9%

Able to read and write English well or very well	63.3%	97.2%	19.3%	56.9%	100%

**Working context§**					

**Operating authority of facility or practice**					

Government	86.3%	59.8%	100%	92.2%	95.8%

Nongovernmental organization	14.5%	26.2%	4.4%	7.8%	21.1%

For-profit organization	9.6%	23.4%	0.7%	0%	12.7%

**Type of facility or practice**					

Solo or individual practice	13.7%	13.1%	9.6%	15.7%	21.4%

Group practice	33.2%	22.4%	53.7%	0%	34.3%

Hospital	81.6%	79.4%	93.4%	51.0%	84.3%

Community health centre	25.0%	20.6%	0.7%	56.9%	55.7%

**Location of facility or practice**					

Urban	16.0%	4.7%	2.4%	31.4%	44.6%

Rural	55.7%	69.8%	60.3%	52.9%	29.7%

Mixed	40.6%	32.1%	45.2%	23.5%	56.8%

Facility had insecticide-treated nets (ITNs) available	58.5%	64.5%	38.5%	54.9%	89.0%

**Views and activities related to improving clinical practice**					

Research performed in their own country is of above average or excellent quality	47.8%	57.3%	28.2%	39.2%	75.3%

Trust somewhat or completely a systematic review of randomized controlled double-blind trials	55.5%	58.7%	48.1%	47.1%	72.6%

Working with researchers or research groups to improve clinical practice or the quality of working life	43.9%	40.6%	41.0%	27.5%	66.2%

Higher quality of available research is important or very important to improve their work	92.6%	86.0%	99.3%	98.0%	86.3%

**Used or read particular sources of evidence**					

Clinical practice guidelines, protocols or decision-support tools	59.4%	65.7%	47.7%	51.0%	80.0%

Cochrane Library	11.1%	20.4%	6.7%	5.9%	9.5%

Scientific journals from high-income countries	35.9%	55.9%	15.6%	29.4%	50.0%

Scientific journals from own country	55.1%	53.6%	64.9%	17.6%	67.2%

Summaries of articles, reports, and reviews from public and not-for-profit health organizations	56.9%	64.1%	36.4%	78.4%	69.2%

§May not add to 100% because health care providers may practise in more than one setting.

†May not add to 100% because the allocation of time reported by a small number of respondents did not add to 100%

Few participating healthcare providers correctly answered all five knowledge questions about ITNs (13%), whether from Ghana (10%), Laos (0%), Senegal (4%) or Tanzania (33%). The overall percentage of correct responses to individual questions ranged from 43% on the effectiveness of torn ITNs (question 1) to 68% on the protective effect of ITNs for non-users in the same house (question 2), with variation greatest within Senegal (8%-98%) and least within Tanzania (70%-85%). Approximately half of all participating healthcare providers knew that using untreated nets can divert extra biting to sleepers without nets in the same houses (48%), that ITNs need regular re-treatment to remain effective while long-lasting ITNs do not (57%), and that ITNs have been demonstrated to reduce the number of malaria episodes in communities with stable malaria (57%) (Table 3).

**Table 3:** Questions assessing healthcare providers' knowledge of malaria prevention

Question (True/False)	All N = 372	Ghana N = 107	Laos N = 136	Senegal N = 51	Tanzania N = 75
Insecticide-treated nets that are torn are no longer effective and should not be used. [*False*]	43.3% (158/365)	32.4% (34/105)	50.7% (69/136)	7.8% (4/51)	69.9% (51/73)

The use of insecticide-treated nets can reduce the number of bites in sleepers without nets in the same houses. [*True*]	68.3% (250/366)	68.6% (72/105)	61.8% (84/136)	78.4% (40/51)	73.0% (54/74)

The use of untreated nets can divert extra biting to sleepers without nets in the same houses. [*True*]	48.5% (176/363)	60.8% (62/102)	25.7% (35/136)	51.0% (26/51)	71.6% (53/74)

Insecticide-treated nets need regular re-treatment to remain effective while long-lasting insecticidal nets remain effective for a long time and after many washes, without the need for re-treatment. [*True*]	57.2% (207/362)	64.7% (66/102)	21.3% (29/136)	98.0% (50/51)	84.9% (62/73)

Insecticide-treated nets' ability to reduce the number of malaria episodes in communities with stable malaria has *not *been demonstrated. [*False*]	56.6% (206/364)	69.5% (73/105)	19.9% (27/136)	88.2% (45/51)	84.7% (61/72)

**All answers correct**	4%	10%	0%	4%	33%

Data show the percentage and fraction of respondents who correctly answered each question

Very few participating healthcare providers self-reported performing all five clinical practices according to established evidence (2%), whether from Ghana (1%), Laos (1%), Senegal (4%) or Tanzania (5%). Approximately half of providers often or very often reported that they enquired about young children's home-use of ITNs (52%), recommended to caregivers that young children use them (56%) and advised caregivers on the need to regularly re-treat them (46%). A quarter of the participating healthcare providers reported often or very often giving ITNs to the caretakers of young children and pregnant women (26%). More than one-third of the respondents self-reported that they often or very often incorrectly informed caretakers that torn ITNs are worse than no ITNs (37%) (Table 4).

**Table 4:** Questions assessing healthcare providers' practices relating to malaria prevention

Question (Frequency)	All N = 372	Ghana N = 107	Laos N = 136	Senegal N = 51	Tanzania N = 75
When treating young children, how often did you enquire about their and their caretakers' home-use of insecticide-treated nets? [*Recommended practice*]	51.9% (191/368)	57.9% (62/107)	41.9% (57/136)	33.3% (17/51)	74.3% (55/74)

When treating young children, how often did you recommend caretakers to use insecticide-treated nets for their young children? [*Recommended practice*]	56.1% (207/369)	65.4% (70/107)	44.9% (61/136)	37.2% (19/51)	76.0% (57/75)

When treating young children, how often did you inform caretakers who used insecticide-treated nets of the need to regularly re-treat their nets? [*Recommended practice*]	46.2% (170/368)	40.2% (43/107)	39.0% (53/136)	37.2% (19/51)	74.3% (55/74)

When treating young children and pregnant women, how often did you (or someone acting on your behalf) provide caretakers and pregnant women with an insecticide-treated nets for home-use? [*Recommended practice*]	26.3% (97/368)	31.1% (33/106)	15.4% (21/136)	33.3% (17/51)	34.7% (26/75)

When treating young children, how often did you inform caretakers that torn insecticide-treated nets are worse than no insecticide-treated nets? [*Contrary to recommended practice*]	36.5% (133/364)	29.5% (31/105)	41.2% (56/136)	35.3% (18/51)	38.9% (28/72)

**All recommended practices**	2%	1%	1%	4%	5%

Data show the percentage and fraction of respondents who over the previous 12 months engaged in the recommended practices described in the first four questions either often or very often (vs. never, rarely, sometimes, and not applicable) and who never engaged in the non-recommended practice as described in the last question (vs. rarely, sometimes, often, very often, and not applicable)

The first ordinal logistic model revealed two statistically significant factors associated with healthcare providers having higher *knowledge *scores related to malaria prevention: 1) training in acquiring systematic reviews through the Cochrane Library since completing their last degree (odds ratio [OR] 2.82, 95% confidence interval [CI] 1.08-7.36); and 2) the ability to read and write English well or very well (OR 1.92, 95% CI 1.37-2.69). The second ordinal logistic model highlighted several factors associated with healthcare providers following better practices relating to malaria prevention, namely: 1) reading scientific journals from their own country (OR 1.67, 95% CI 1.10-2.54); 2) working with researchers to improve their clinical practice or quality of working life (OR 1.44, 95% CI 1.04-1.98); 3) training on malaria prevention since their last degree (OR 1.68, 95% CI 1.17-2.39); and 4) easy access to the internet (OR 1.52, 95% CI 1.08-2.14) (Table 5).

**Table 5:** Ordinal logistic models for the factors associated with the log odds of having higher knowledge and better practices

Factor	Knowledge (n = 340)		Practices (n = 340)	
	**OR**	**95% CI**	**OR**	**95% CI**

**Individual characteristics**				

Age*	1.07	(0.96, 1.19)	1.14	(0.94, 1.38)

Age squared*	1.00	(1.00, 1.00)	1.00	(1.00, 1.00)

Sex, male	1.16	(0.93, 1.46)	1.28	(0.98, 1.68)

Specialist physician	1.15	(0.60, 2.20)	1.82	(0.71, 4.65)

Time allocated to research **	1.01	(1.00, 1.01)	1.01	(0.99, 1.02)

Master's or doctorate degree	1.01	(0.81, 1.26)	0.81	(0.48, 1.35)

Training (since completed last degree) in:				

Acquiring systematic reviews through the Cochrane Library	**2.82**	**(1.08, 7.36)**	0.99	(0.49, 2.00)

Critically appraising systematic reviews	0.77	(0.25, 2.40)	1.28	(0.66, 2.47)

Integrated Management of Childhood Illness (IMCI)	0.72	(0.41, 1.28)	0.95	(0.48, 1.90)

Prevention of malaria	1.20	(0.73, 1.97)	**1.68**	**(1.17, 2.39)**

Easy access to a personal computer with a CD ROM	1.50	(0.63, 3.58)	0.63	(0.28, 1.43)

Easy access to the internet	1.07	(0.44, 2.57)	**1.52**	**(1.08, 2.14)**

Able to read and write English well or very well	**1.92**	**(1.37, 2.69)**	1.00	(0.63, 1.58)

**Working Context**				

Based in a facility or practice with an NGO as the operating authority	0.90	(0.32, 2.53)	1.25	(0.66, 2.37)

Based in a hospital	1.08	(0.83, 1.39)	0.66	(0.32, 1.34)

Located in an urban setting	1.01	(0.85, 1.19)	1.00	(0.73, 1.37)

Facility had insecticide-treated nets (ITNs) available	0.71	(0.35, 1.46)	2.58	(0.52, 12.94)

**Views and activities related to improving clinical practice**				

Research performed in their own country is of above average or excellent quality	0.78	(0.35, 1.73)	1.49	(0.88, 2.53)

Trust somewhat or completely a systematic review of randomized controlled double-blind trials	1.06	(0.63, 1.79)	1.16	(0.52, 2.58)

Working with researchers or research groups to improve clinical practice or the quality of working life	1.11	(0.69, 1.78)	**1.44**	**(1.04, 1.98)**

Higher quality of available research is important or very important to improve their work	1.07	(0.54, 2.14)	0.63	(0.33, 1.18)

**Used or read particular sources of evidence**				

Clinical practice guidelines, protocols or decision-support tools	1.06	(0.71, 1.60)	1.32	(0.83, 2.10)

Cochrane Library	0.68	(0.37, 1.27)	0.98	(0.56, 1.70)

Scientific journals from high-income countries	1.09	(0.50, 2.36)	0.74	(0.47, 1.16)

Scientific journals from own country	1.31	(0.88, 1.95)	**1.67**	**(1.10, 2.54)**

Summaries of articles, reports, and reviews from public and not-for-profit health organizations	1.37	(0.50, 3.75)	1.27	(0.92, 1.76)

*Thresholds*				

k^1^	1.81	(-0.29, 3.91)	2.59	(-2.31, 7.49)

k^2^	3.03	(1.23, 4.84)	3.87	(-0.99, 8.73)

k^3^	3.33	(1.16, 5.49)	4.99	(0.16, 9.81)

k^4^	4.59	(1.90, 7.28)	5.92	(0.63, 11.21)

CI = confidence interval, NGO = nongovernmental organization, OR = odds ratio

Standard errors adjusted for 4 clusters (i.e., country). All regression models include country dummies (Tanzania is the reference country)

* Entered in regression models as continuous variables measured in years

** Entered in regression models as continuous variable measured in percent of time

## Discussion

### Principal findings

There is room for improvement in the knowledge and practices relevant to the prevention of malaria among the healthcare providers in low- and middle-income countries that were included in this study. Many misconceptions of ITNs remain common even among highly educated healthcare providers. For example, many participating healthcare providers did not know that the use of untreated nets can divert extra biting to sleepers without nets in the same home, and many of them self-reported that they often (incorrectly) informed patients that torn ITNs are worse than no ITNs. These gaps in knowledge and practices on the prevention of malaria align with other gaps that have been well-documented among households on the same issue [16-39] and healthcare providers on the diagnosis, treatment and transmission of malaria in low- and middle-income countries [45-50] and other diseases and conditions around the world [51-73,101-122].

This study also found various alterable factors that were associated with higher knowledge and better practices. For example, healthcare providers that had access to the internet and training in malaria prevention reported better practices relating to malaria prevention than those who did not. This study also indicates that healthcare providers who read journals from their own country not only *self-report *that these journals lead to changes in their clinical practice, as previous studies have found [75,80], but that reading these journals is actually associated with better self-reported practices. In addition, this study confirms the importance of interactions between healthcare providers and researchers for achieving evidence-based practice as has been widely reported in the research literature [123,124]. This study further offers some data and a baseline to help define the scope of this challenge.

The logistic models suggest that higher knowledge and better practices may be associated with different factors. While it may be the case that these factors influence knowledge and practices differently, these divergent results could also be explained by social desirability bias which presumably would affect self-reports of practices much more than it would affect answers to knowledge-testing questions. Alternatively, the models may lack sufficient power, the dependent variables may not adequately reflect respondents' real "knowledge" and "practice", or there may be confounding variables affecting the analysis.

### Policy implications

Improving healthcare providers' knowledge and practices is an untapped opportunity for expanding ITN coverage and preventing malaria. Not only are healthcare providers the main source through which people learn about and use health interventions [1], but they are known to greatly influence their patients' health-related behaviours [40-44]. Now that gaps in knowledge and practices relating to malaria prevention have been identified, appropriate interventions targeting healthcare providers can be deployed with the possibility that small investments directed at this influential group may efficiently achieve significant returns across large populations. These interventions can be informed by the many studies conducted on how to bring about behaviour change among healthcare providers, including systematic reviews of studies on the effectiveness of audit and feedback [125], distribution of education materials [126], educational meetings [127], local opinion leaders [128], outreach visits [91], and reminders [129], as well as the Rx for Change and Health Systems Evidence databases which contain these types of reviews [130,131]. In addition, the results from this study also highlight several changeable factors that may be helpful for improving healthcare providers' knowledge and practices more broadly. This is information that national health policymakers, civil society leaders, donors and international organizations can use to design optimized strategies and evidence-informed policies that may lead to accelerated progress in this area.

Specifically, this study's results point to several possible areas in which interventions may be effective in helping healthcare providers attain greater knowledge on malaria prevention and evidence-based practices. First, the low proportion of healthcare providers who received training in acquiring, assessing and adapting research evidence suggests such training is an opportunity for improvement--especially if it covers systematic reviews. Indeed, whereas graduate degrees and clinical specialization were not associated with greater knowledge on malaria prevention, training in acquiring systematic reviews through the Cochrane Library was. Second, improving access to computers and the internet may be fruitful. While only 11% of the total sample used the Cochrane Library (which is free in most low-income countries), this doubles to 25% for those who have easy access to the internet. Third, the fact that English language abilities were associated with higher knowledge suggests that the best health knowledge and/or training opportunities may not be available to healthcare providers in their local languages. Efforts to disseminate translated research syntheses may be helpful. Fourth, supporting interaction between healthcare providers and researchers may also be an effective strategy for improving clinical practice. Finally, access to scientific journals from healthcare providers' own country can be facilitated, especially as the studies they contain would be among the most applicable to their local context.

### Strengths and limitations of the study

This study has five main strengths. First, it examines malaria, a disease prioritized in the Millennium Development Goals and which has a proven intervention for prevention that is not universally utilized (i.e., ITNs). Second, the study's questionnaire was built on existing questionnaires, pilot tested, and assessed for reliability and validity [74]. Third, data were collected from four low- and lower-middle-income countries with different life expectancies, coverage rates for ITNs, and other characteristics. Fourth, high response rates were achieved in three of four countries. Fifth, knowledge and practice scores were calculated from a range of testing questions for which the participating healthcare providers were not told the correct answer, which is a more objective metric than asking participants to self-evaluate whether they had "high" or "low" knowledge and practices as has been done in past studies.

This study has four main limitations. First, the survey relies on self-reported data to assess healthcare providers' practices which increases the risk of social desirability bias and may differ from actual performance. One review of studies conducted between 1980-1996 suggests self-reports of practices overestimate actual behaviour (in that case, of adherence to practice guidelines) by up to 27% [132]. Second, the composite knowledge and practice scores were based on responses to only ten questions. Third, linguistic and cultural differences may have affected respondents' interpretation of certain questions. Fourth, resource constraints prevented the survey of fully representative samples of healthcare providers in the four countries which means results cannot and should not be compared across countries. For example, participants in Tanzania were mostly district or regional medical officers who were attending a meeting on malaria--a group that would be expected to have relative good knowledge and practices--whereas the majority of respondents in Senegal were not physicians given this country's reliance on nurses and community health workers for preventative care. Future studies like this one, if possible, should be conducted using representative samples in order to enhance generalizability.

## Conclusions

Increased attention is necessary to help bridge the gap between what is known from research evidence about preventing malaria and the knowledge and practices of healthcare providers in low- and middle-income countries. Until serious and multi-pronged efforts are implemented, we may never be able to fully capitalize on existing evidence-based and cost-effective interventions like ITNs for reducing the burden of malaria, addressing inequities and improving health globally. Those policymakers, civil society leaders, donors and international organizations who are working to prevent malaria should at least consider strategies to enhance healthcare provider' knowledge and evidence-based practice, such as offering training on acquiring systematic reviews, facilitating access to the internet, translating research syntheses to local languages, encouraging interactions with researchers, making ITNs available at healthcare facilities, and supporting the dissemination of local scientific journals. Further studies are necessary to both confirm these exploratory findings and more precisely inform future policy directions.

## Competing interests

The authors declare that they have no competing interests.

## Authors' contributions

SJH, GEG, JNL, GDN, EJAO, MFS and BB contributed substantially to the study concept and design, the acquisition of data, or the analysis and interpretation of data, and revised the article critically for important intellectual content. SJH led the drafting of the paper, GEG led the data analysis, and JNL led the overall study design. All authors gave their approval for this paper to be published.

## Funding

Funding for this project was provided by the Alliance for Health Policy & Systems Research and the Global Development Network and supplemented with substantial in-kind support of staff time and other resources from McMaster University and the World Health Organization. John Lavis receives salary support as the Canada Research Chair in Knowledge Transfer and Exchange. The views expressed in this paper are those of the authors and do not represent the views of their affiliated organizations.

## References

[B1] Haines A Kuruvilla S Borchert M Bridging the implementation gap between knowledge and action for health Bull World Health Organ 2004 82 724 731 15643791 PMC2623035

[B2] World Health Organization World report on knowledge for better health: Strengthening health systems 2004 Geneva: World Health Organization

[B3] Jones G Steketee RW Black RE Bhutta ZA Morris SS Bellagio Child Survival Study Group How many child deaths can we prevent this year? Lancet 2003 362 65 71 10.1016/S0140-6736(03)13811-1 12853204

[B4] Lengeler C Insecticide-treated bed nets and curtains for preventing malaria Cochrane Database Syst Rev 2004 CD000363 10.1002/14651858.CD000363.pub2 15106149

[B5] Gamble C Ekwaru JP ter Kuile FO Insecticide-treated nets for preventing malaria in pregnancy Cochrane Database Syst Rev 2006 CD003755 10.1002/14651858.CD003755.pub2 PMC6532581 16625591

[B6] Gamble C Ekwaru PJ Garner P ter Kuile FO Insecticide-treated nets for the prevention of malaria in pregnancy: a systematic review of randomised controlled trials PLoS Med 2007 4 e107 10.1371/journal.pmed.0040107 17388668 PMC1831739

[B7] Roll Back Malaria Partnership The Global Malaria Action Plan: For a Malaria-Free World 2008 Geneva: Roll Back Malaria Partnership

[B8] Teklehaimanot A McCord GC Sachs JD Scaling up malaria control in Africa: an economic and epidemiological assessment Am J Trop Med Hyg 2007 77 138 144 18165486

[B9] Snow RW Guerra CA Mutheu JJ Hay SI International funding for malaria control in relation to populations at risk of stable *Plasmodium falciparum *transmission PLoS Med 2008 7 e142 10.1371/journal.pmed.0050142 PMC2488181 18651785

[B10] Snow RW Marsh K Malaria in Africa: progress and prospects in the decade since the Abuja Declaration Lancet 2010 376 137 139 10.1016/S0140-6736(10)60577-6 20417552 PMC2907486

[B11] World Health Organization World Malaria Report 2009 2009 Geneva: World Health Organization

[B12] World Health Assembly WHA58.2: Malaria Control. Fifty-Eighth World Health Assembly 2005 World Health Organization. Geneva

[B13] World Bank The Millennium Development Goals for Health: Rising to the Challenges 2004 Washington DC: World Bank

[B14] Bhattarai A Ali AS Kachur SP Martensson A Abbas AK Khatib R Al-Mafazy AW Ramsan M Rotllant G Gerstenmaier JF Molteni F Abdulla S Montgomery SM Kaneko A Bjorkman A Impact of artemisinin-based combination therapy and insecticide-treated nets on malaria burden in Zanzibar PLoS Med 2007 4 e309 10.1371/journal.pmed.0040309 17988171 PMC2062481

[B15] United Nations The Millennium Development Goals Report 2009 2009 New York: United Nations

[B16] Deressa W Ali A Enquoselassie F Knowledge, attitude and practice about malaria the mosquito and antimalarial drugs in a rural community Ethiopian J Health Dev 2003 17 99 104

[B17] Ahorlu CK Dunyo SK Afari EA Koram KA Nkrumah FK Malaria-related beliefs and behaviour in southern Ghana: implications for treatment, prevention and control Trop Med Int Health 1997 2 488 499 10.1111/j.1365-3156.1997.tb00172.x 9217705

[B18] NetMark NetMark 2004 survey on insecticide-treated nets (ITNs) in Ghana 2005 Washington DC: NetMark

[B19] Nieto T Mendez F Carrasquilla G Knowledge, beliefs and practices relevant for malaria control in an endemic urban area of the Colombian Pacific Soc Sci Med 1999 49 601 609 10.1016/S0277-9536(99)00134-3 10452416

[B20] Simsek Z Kurcer MA Malaria: knowledge and behaviour in an endemic rural area of Turkey Public Health 2005 119 202 208 10.1016/j.puhe.2004.03.011 15661131

[B21] Pistone T Guibert P Gay F Malvy D Ezzedine K Receveur MC Siriwardana M Larouze B Bouchaud O Malaria risk perception, knowledge and prophylaxis practices among travellers of African ethnicity living in Paris and visiting their country of origin in sub-Saharan Africa Trans R Soc Trop Med Hyg 2007 101 990 995 10.1016/j.trstmh.2007.05.009 17643457

[B22] Mwenesi H Harpham T Snow RW Child malaria treatment practices among mothers in Kenya Soc Sci Med 1995 40 1271 1277 10.1016/0277-9536(94)00250-W 7610432

[B23] Espino F Manderson L Acuin C Domingo F Ventura E Perceptions of malaria in a low endemic area in the Philippines: transmission and prevention of disease Acta Trop 1997 63 221 239 10.1016/S0001-706X(96)00623-7 9088436

[B24] Njama D Dorsey G Guwatudde D Kigonya K Greenhouse B Musisi S Kamya MR Urban malaria: primary caregivers' knowledge, attitudes, practices and predictors of malaria incidence in a cohort of Ugandan children Trop Med Int Health 2003 8 685 692 10.1046/j.1365-3156.2003.01060.x 12869089

[B25] Kemble SK Davis JC Nalugwa T Njama-Meya D Hopkins H Dorsey G Staedke SG Prevention and treatment strategies used for the community management of childhood fever in Kampala, Uganda Am J Trop Med Hyg 2006 74 999 1007 16760510

[B26] Miguel CA Tallo VL Manderson L Lansang MA Local knowledge and treatment of malaria in Agusan del Sur, The Philippines Soc Sci Med 1999 48 607 618 10.1016/S0277-9536(98)00352-9 10080362

[B27] Cropley L The effect of health education interventions on child malaria treatment-seeking practices among mothers in rural refugee villages in Belize, Central America Health Promot Int 2004 19 445 452 10.1093/heapro/dah406 15520038

[B28] Sanjana P Barcus MJ Bangs MJ Ompusunggu S Elyazar I Marwoto H Tuti S Sururi M Tjokrosonto S Baird JK Survey of community knowledge, attitudes, and practices during a malaria epidemic in central Java, Indonesia Am J Trop Med Hyg 2006 75 783 789 17123966

[B29] Dulhunty JM Yohannes K Kourleoutov C Manuopangai VT Polyn MK Parks WJ Bryan JH Malaria control in central Malaita, Solomon Islands 2. Local perceptions of the disease and practices for its treatment and prevention Acta Trop 2000 75 185 196 10.1016/S0001-706X(00)00057-7 10708658

[B30] Yohannes K Dulhunty JM Kourleoutov C Manuopangai VT Polyn MK Parks WJ Williams GM Bryan JH Malaria control in central Malaita, Solomon Islands. 1. The use of insecticide-impregnated bed nets Acta Trop 2000 75 173 183 10.1016/S0001-706X(00)00055-3 10708657

[B31] Kengeya-Kayondo JF Seeley JA Kajura-Bajenja E Kabunga E Mubiru E Sembajja F Mulder DW Recognition, treatment seeking behaviour and perception of cause of malaria among rural women in Uganda Acta Trop 1994 58 267 273 10.1016/0001-706X(94)90020-5 7709865

[B32] Schultz LJ Ettling M Chitsulo L Steketee RW Nyasulu Y Macheso A Nwanyanwu OC A nation-wide malaria knowledge, attitudes and practices survey in Malawi: objectives and methodology Trop Med Parasitol 1994 45 54 56 8066386

[B33] Adera TD Beliefs and traditional treatment of malaria in Kishe settlement area, southwest Ethiopia Ethiop Med J 2003 41 25 34 12764998

[B34] Keating J Eisele TP Bennett A Johnson D Macintyre K A description of malaria-related knowledge, perceptions, and practices in the Artibonite Valley of Haiti: implications for malaria control Am J Trop Med Hyg 2008 78 262 269 18256427

[B35] Oguonu T Okafor H Obu H Caregivers' knowledge, attitude and practice on childhood malaria and treatment in urban and rural communities in Enugu, south-east Nigeria Public Health 2005 119 409 414 10.1016/j.puhe.2004.05.008 15780330

[B36] Govere J Durrheim D La Grange K Mabuza A Booman M Community knowledge and perceptions about malaria and practices influencing malaria control in Mpumalanga province, South Africa S Afr Med J 2000 90 611 616 10918892

[B37] Hlongwana K Mabaso M Kunene S Govender D Maharaj R Community knowledge, attitudes and practices (KAP) on malaria in Swaziland: A country earmarked for malaria elimination Malar J 2009 8 29 10.1186/1475-2875-8-29 19228387 PMC2649151

[B38] Paulander J Olsson H Lemma H Getachew A San Sebastian M Knowledge, attitudes and practice about malaria in rural Tigray, Ethiopia Global Health Action 2009 2 10.3402/gha.v2i0.1839 PMC2779931 20027277

[B39] Gingrich CD Hanson KG Marchant TJ Mulligan J-A Mponda H Household demand for insecticide-treated bednets in Tanzania and policy options for increasing uptake Health Policy Plan 2011 26 133 141 10.1093/heapol/czq027 20660208

[B40] Stead LF Bergson G Lancaster T Physician advice for smoking cessation Cochrane Database Syst Rev 2008 CD000165 10.1002/14651858.CD000165.pub3 18425860

[B41] Rice VH Stead LF Nursing interventions for smoking cessation Cochrane Database Syst Rev 2008 CD001188 10.1002/14651858.CD001188.pub3 18253987

[B42] Sinclair HK Bond CM Stead LF Community pharmacy personnel interventions for smoking cessation Cochrane Database Syst Rev 2004 CD003698 10.1002/14651858.CD003698.pub2 14974031

[B43] Brunner EJ Rees K Ward K Burke M Thorogood M Dietary advice for reducing cardiovascular risk Cochrane Database Syst Rev 2007 CD002128 10.1002/14651858.CD002128.pub4 23543514

[B44] Hooper L Bartlett C Davey SG Ebrahim S Advice to reduce dietary salt for prevention of cardiovascular disease Cochrane Database Syst Rev 2004 CD003656 10.1002/14651858.CD003656.pub2 PMC12160996 14974027

[B45] Onwujekwe O Uzochukwu B Dike N Uguru N Nwobi E Shu E Malaria treatment perceptions, practices and influences on provider behaviour: comparing hospitals and non-hospitals in south-east Nigeria Malar J 2009 8 246 10.1186/1475-2875-8-246 19863803 PMC2775747

[B46] Smith LA Jones C Meek S Webster J Provider practice and user behavior interventions to improve prompt and effective treatment of malaria: do we know what works? Am J Trop Med Hyg 2009 80 326 335 19270276

[B47] Mayxay M Pongvongsa T Phompida S Phetsouvanh R White NJ Newton PN Diagnosis and management of malaria by rural community health providers in the Lao People's Democratic Republic (Laos) Trop Med Int Health 2007 12 540 546 10.1111/j.1365-3156.2007.01820.x 17445145

[B48] Wagbatsoma VA Ogbeide O Towards malaria control: the knowledge of health care providers about mosquito and malaria transmission Cent Afr J Med 1999 45 4 6 10444888 10.4314/cajm.v45i1.8441

[B49] Nahum A Akogbeto M [Malaria and pregnancy: attitude of health care personnel during prenatal care in Cotonou, Benin] Med Trop (Mars) 2000 60 251 255 11258057

[B50] Faye O Ndir O Gaye O Bah IB Dieng T Dieng Y Diallo S Diagne AK [Health personnel and population practices in the diagnosis of malaria and use of antimalarial drugs in Dakar] Med Trop (Mars) 1995 55 47 50 7637609

[B51] Studts JL Burris JL Kearns DK Worth CT Sorrell CL "Providers practice prevention": promoting dental hygienists' use of evidence-based treatment of tobacco use and dependence J Dent Educ 2009 73 1069 1082 19734248

[B52] Sheffer CE Barone CP Anders ME Training health care providers in the treatment of tobacco use and dependence: pre- and post-training results J Eval Clin Pract 2009 15 607 613 10.1111/j.1365-2753.2008.01058.x 19674215

[B53] Applegate BW Sheffer CE Crews KM Payne TJ Smith PO A survey of tobacco-related knowledge, attitudes and behaviours of primary care providers in Mississippi J Eval Clin Pract 2008 14 537 544 10.1111/j.1365-2753.2007.00910.x 18462288

[B54] Bigelow C Patton LL Strauss RP Wilder RS North Carolina dental hygienists' view on oral cancer control J Dent Hyg 2007 81 83 18173897

[B55] Jenkins K Ahijevych K Nursing students' beliefs about smoking, their own smoking behaviors, and use of professional tobacco treatment intervention Appl Nurs Res 2003 16 164 172 10.1016/S0897-1897(03)00047-8 12931330

[B56] Menees SB Patel DA Dalton V Colorectal cancer screening practices among obstetrician/gynecologists and nurse practitioners J Women's Health (Larchmont) 2009 18 1233 1238 10.1089/jwh.2008.1117 19630544

[B57] Rim SH Zittleman L Westfall JM Overholser L Froshaug D Coughlin SS Knowledge, attitudes, beliefs, and personal practices regarding colorectal cancer screening among health care professionals in rural Colorado: a pilot survey J Rural Health 2009 25 303 308 10.1111/j.1748-0361.2009.00234.x 19566617

[B58] Berkowitz Z Hawkins NA Peipins LA White MC Nadel MR Beliefs, risk perceptions, and gaps in knowledge as barriers to colorectal cancer screening in older adults J Am Geriatr Soc 2008 56 307 314 10.1111/j.1532-5415.2007.01547.x 18070002

[B59] Sabatino SA McCarthy EP Phillips RS Burns RB Breast cancer risk assessment and management in primary care: provider attitudes, practices, and barriers Cancer Detect Pre J 2007 31 375 383 10.1016/j.cdp.2007.08.003 18037249

[B60] Daley MF Liddon N Crane LA Beaty BL Barrow J Babbel C Markowitz LE Dunne EF Stokley S Dickinson LM Berman S Kempe A A national survey of pediatrician knowledge and attitudes regarding human papillomavirus vaccination Pediatrics 2006 118 2280 2289 10.1542/peds.2006-1946 17142510

[B61] Nizami SQ Khan IA Bhutta ZA Differences in self-reported and observed prescribing practice of general practitioners and paediatricians for acute watery diarrhoea in children of Karachi, Pakistan J Diarrhoeal Dis Res 1995 13 29 32 7657962

[B62] Kundi MZ Ahmad I Anjum M Evaluation of diarrhoea management of health professionals trained at the Diarrhoea Training Unit of Rawalpindi General Hospital J Pak Med Assoc 1997 47 3 6 9056728

[B63] Ibrahim S Isani Z Evaluation of doctors trained at Diarrhoea Training Unit of National Institute of Child Health, Karachi J Pak Med Assoc 1997 47 7 11 9056729

[B64] Kasi M Kausar P Naz R Miller LC Treatment of diarrhoea in infants by medical doctors in Balochistan, Pakistan J Diarrhoeal Dis Res 1995 13 238 241 8838828

[B65] Singh J Bora D Sachdeva V Sharma RS Verghese T Prescribing pattern by doctors for acute diarrhoea in children in Delhi, India J Diarrhoeal Dis Res 1995 13 229 231 8838825

[B66] Raghu MB Balasubramanian S Indumathy Balasubrahmanyam G Awareness of and attitude towards oral rehydration therapy Indian J Pediatr 1995 62 439 443 10.1007/BF02755064 10829902

[B67] Ozuah PO Avner JR Stein RE Oral rehydration, emergency physicians, and practice parameters: a national survey Pediatrics 2002 109 259 261 10.1542/peds.109.2.259 11826204

[B68] Choudhry AJ Mubasher M Factors influencing the prescribing patterns in acute watery diarrhoea J Pak Med Assoc 1997 47 32 35 9056735

[B69] Steiner MJ Raymond E Attafuah JD Hays M Provider knowledge about emergency contraception in Ghana J Biosoc Sci 2000 32 99 106 10.1017/S0021932000000997 10676062

[B70] Suhaimi H Monga D Siva A A study of knowledge and attitudes towards contraception among health care staff in Kelantan (Malaysia) Singapore Med J 1996 37 51 54 8783914

[B71] Khasiani SA Family planning knowledge, attitudes and practices among health centre personnel in Western Province of Kenya J Obstet Gynaecol East Cent Afr 1991 9 30 36 12316814

[B72] Quereshi ZP Sekadde-kigondu CB A survey to determine the knowledge, attitudes and practice of family planning amongst the nursing staff at Kenyatta National Hospital J Obstet Gynaecol East Cent Afr 1991 9 49 51 12316816

[B73] Gaffikin L Phiri A McGrath J Zinanga A Blumenthal PD Provider attitudes toward IUD provision in Zimbabwe: perception of HIV risk and training implications Adv Contracept 1998 14 27 39 10.1023/A:1006519409808 9587006

[B74] Guindon GE Lavis J Boupha B Shi G Sidibe M Turdaliyeva B Research to Policy and Practice Study Team Bridging the gaps among research, policy and practice in ten low- and middle-income countries: Development and testing of a questionnaire for health-care providers Health Res Policy Syst 2010 8 3 20205838 10.1186/1478-4505-8-3 PMC2825186

[B75] Guindon GE Lavis JN Becerra-Posada F Malek-Afzali H Shi G Yesudian CAK Hoffman SJ for the Research to Policy and Practice Study Team Bridging the gaps between research, policy and practice in low- and middle-income countries: a survey of health care providers Can Med Assoc J 2010 182 E362 372 10.1503/cmaj.081165 PMC2882467 20439448

[B76] Cameron D Lavis JN Guindon GE Akhtar T Becerra Posada F Ndossi G Boupha B Research to Policy and Practice Study Team Bridging the gaps among research, policy and practice in ten low- and middle-income countries: Development and testing of a questionnaire for researchers Health Res Policy Syst 2010 8 4 20205837 10.1186/1478-4505-8-4 PMC2825187

[B77] Lavis JN Guindon GE Cameron D Boupha B Dejman M Osei EJA Sadana R for the Research to Policy and Practice Study Team Bridging the gaps between research, policy and practice in low- and middle-income countries: a survey of researchers Can Med Assoc J 2010 182 E350 361 10.1503/cmaj.081164 PMC2882466 20439449

[B78] McColl A Smith H White P Field J General practitioner's perceptions of the route to evidence based medicine: a questionnaire survey BMJ 1998 316 361 365 10.1136/bmj.316.7128.361 9487174 PMC2665572

[B79] Incorporated MI Demographic and Health Surveys: The Service Provision Assessment 2004 Calverton, Maryland: Macro International Incorporated

[B80] Page J Heller RF Kinlay S Lim LL Qian W Suping Z Kongpatanakul S Akhtar M Khedr S Macharia W Attitudes of developing world physicians to where medical research is performed and reported BMC Public Health 2003 3 6 10.1186/1471-2458-3-6 12529182 PMC149227

[B81] Prescott K Lloyd M Douglas HR Haines A Humphrey C Rosenthal J Watt I Promoting clinically effective practice: general practitioners' awareness of sources of research evidence Fam Pract 1997 14 320 323 10.1093/fampra/14.4.320 9283854

[B82] Wilson P Droogan J Glanville J Watt I Hardman G Access to the evidence base from general practice: a survey of general practice staff in Northern and Yorkshire Region Qual Saf Health Care 2001 10 83 89 10.1136/qhc.10.2.83 PMC1757985 11389316

[B83] Wilson P Glanville J Watt I Access to the online evidence base in general practice: a survey of the Northern and Yorkshire Region Health Inf Libr J 2003 20 172 178 10.1046/j.1365-2532.2003.00448.x 12919280

[B84] World Health Organization WHO Health Research Utilization Assessment Project: Questionnaire for Health Providers -Pilot 2002 Principal investigator: Shyama Kuruvilla. Geneva: World Health Organization

[B85] World Health Organization Integrated Management of Childhood Illness (IMCI) Multi-Country Evaluation -Health Facility Survey 2004 Geneva: World Health Organization

[B86] Landry R Lamari M Amara N The extent and determinants of the utilization of university research in government agencies Public Adm Rev 2003 63 192 205 10.1111/1540-6210.00279

[B87] Lengeler C, Cattani J, Savigny D Net Gain: Operational Aspects of a New Health Intervention for Preventing Malaria Death 1997 Geneva and Ottawa: World Health Organization and International Development Research Centre

[B88] Lines JD Myamba J Curtis CF Experimental hut trials of permethrin-impregnated mosquito nets and eave curtains against malaria vectors in Tanzania Med Vet Entomol 1987 1 37 51 10.1111/j.1365-2915.1987.tb00321.x 2979519

[B89] Maxwell CA Chambo W Mwaimu M Magogo F Carneiro IA Curtis CF Variation of malaria transmission and morbidity with altitude in Tanzania and with introduction of alphacypermethrin treated nets Malar J 2003 2 28 10.1186/1475-2875-2-28 14585106 PMC239954

[B90] Tami A Mubyazi G Talbert A Mshinda H Duchon S Lengeler C Evaluation of Olyset insecticide-treated nets distributed seven years previously in Tanzania Malar J 2004 3 19 10.1186/1475-2875-3-19 15225349 PMC455684

[B91] United Nations Population Division World Population Prospects: The 2004 Revision 2005 New York: United Nations

[B92] International Monetary Fund World Economic Outlook Database 2007 Washington DC: International Monetary Fund

[B93] World Health Organization World Health Statistics 2007 2007 Geneva: World Health Organization

[B94] United Nations Statistics Division United Nations Common Database 2008 New York: United Nations

[B95] United Nations Children's Fund The State of the World's Children 2008 2007 New York: United Nations22184598

[B96] StataCorp Stata Multiple Imputation Reference Manual Release 11 2009 College Station, Texas: Stata Press

[B97] Cook DJ Mulrow CD Haynes RB Systematic reviews: synthesis of best evidence for clinical decisions Ann Intern Med 1997 126 376 380 9054282 10.7326/0003-4819-126-5-199703010-00006

[B98] Lavis JN Davies HT Gruen RL Walshe K Farquhar CM Working within and beyond the cochrane collaboration to make systematic reviews more useful to healthcare managers and policy makers Healthcare Policy 2006 1 21 33 19305650 PMC2585325

[B99] Oxman AD Guyatt G Guyatt G When to Believe a Subgroup Analysis Users' Guide to the Medical Literature: A Manual for Evidence-Based Clinical Practice 2002 Chicago: AMA Press 553 56522184716

[B100] Haynes RB Mulrow CD Huth EJ Altman DG Gardner MJ More informative abstracts revisited Ann Intern Med 1990 113 69 76 2190518 10.7326/0003-4819-113-1-69

[B101] Gigerenzer G, Gray JAM Better Doctors, Better Patients, Better Decisions: Envisioning Health Care 2020 2011 Cambridge, MA: MIT Press

[B102] Zonfrillo MR Nelson KA Durbin DR Emergency physicians' knowledge and provision of child passenger safety information Acad Emerg Med 2011 18 145 151 10.1111/j.1553-2712.2010.00971.x 21314773

[B103] Shih YC Sleath BL Health care provider knowledge of drug formulary status in ambulatory care settings Am J Health Syst Pharm 2004 61 2657 2663 15646700 10.1093/ajhp/61.24.2657

[B104] Hodges B Inch C Silver I Improving the psychiatric knowledge, skills, and attitudes of primary care physicians, 1950-2000: a review Am J Psychiatry 2001 158 1579 1586 10.1176/appi.ajp.158.10.1579 11578983

[B105] Parada JP Schwartz DN Schiff GD Weiss KB Effects of type and level of training on variation in physician knowledge in the use and acquisition of blood cultures: a cross sectional survey BMC Infect Dis 2005 5 71 10.1186/1471-2334-5-71 16164757 PMC1261264

[B106] Soons KR Lynch T Seagrave M Rolley L Determining physicians' knowledge and attitudes when prescribing drugs to treat gastrointestinal disorders Clin Perform Qual Health Care 1997 5 94 98 10167220

[B107] Mona Eng P Seeger JD Loughlin J Oh K Walker AM Serum potassium monitoring for users of ethinyl estradiol/drospirenone taking medications predisposing to hyperkalemia: physician compliance and survey of knowledge and attitudes Contraception 2007 75 101 107 10.1016/j.contraception.2006.08.011 17241838

[B108] Volpe M Dedhiya SD Physicians, patients, and public knowledge and perception regarding hypertension and stroke: a review of survey studies Curr Med Res Opin 2006 22 1319 1330 10.1185/030079906X112570 16834831

[B109] Zickmund SL Brown KE Bielefeldt K A systematic review of provider knowledge of hepatitis C: is it enough for a complex disease? Dig Dis Sci 2007 52 2550 2556 10.1007/s10620-007-9753-0 17406823

[B110] Zeteroglu S Sahin G Sahin HA Bolluk G Knowledge and attitudes towards emergency contraception of health-care providers in a region with a high birth rate Eur J Contracept Reprod Health Care 2004 9 102 106 10.1080/13625180410001715672 15449822

[B111] Krille L Hammer GP Merzenich H Zeeb H Systematic review on physician's knowledge about radiation doses and radiation risks of computed tomography Eur J Radiol 2010 76 36 41 10.1016/j.ejrad.2010.08.025 20837382

[B112] Wurst KE Sleath BL Physician knowledge and adherence to prescribing antibiotic prophylaxis for sickle cell disease Int J Qual Health Care 2004 16 245 251 10.1093/intqhc/mzh033 15150156

[B113] Kimura S Pacala JT Pressure ulcers in adults: family physicians' knowledge, attitudes, practice preferences, and awareness of AHCPR guidelines J Fam Pract 1997 44 361 368 9108833

[B114] Chin MH Friedmann PD Cassel CK Lang RM Differences in generalist and specialist physicians' knowledge and use of angiotensin-converting enzyme inhibitors for congestive heart failure J Gen Intern Med 1997 12 523 530 10.1046/j.1525-1497.1997.07105.x 9294785 PMC1497156

[B115] Linnehan MJ Andrews S Groce NE College health providers' knowledge, attitudes, and management practices of genital HPV infection Nurse Pract 1996 21 122 124 127-129 8734630

[B116] van Gerwen M Franc C Rosman S Le Vaillant M Pelletier-Fleury N Primary care physicians' knowledge, attitudes, beliefs and practices regarding childhood obesity: a systematic review Obesity Rev 2009 10 227 236 10.1111/j.1467-789X.2008.00532.x 19021874

[B117] Wolfert MZ Gilson AM Dahl JL Cleary JF Opioid analgesics for pain control: wisconsin physicians' knowledge, beliefs, attitudes, and prescribing practices Pain Med 2010 11 425 434 10.1111/j.1526-4637.2009.00761.x 20002590

[B118] Socolar RR Raines B Chen-Mok M Runyan DK Green C Paterno S Intervention to improve physician documentation and knowledge of child sexual abuse: a randomized, controlled trial Pediatrics 1998 101 817 824 10.1542/peds.101.5.817 9565408

[B119] Chen DT Wynia MK Moloney RM Alexander GC U.S. physician knowledge of the FDA-approved indications and evidence base for commonly prescribed drugs: results of a national survey Pharmacoepidemiol Drug Saf 2009 18 1094 1100 10.1002/pds.1825 19697444

[B120] Carr VJ Faehrmann C Lewin TJ Walton JM Reid AA Determining the effect that consultation-liaison psychiatry in primary care has on family physicians' psychiatric knowledge and practice Psychosomatics 1997 38 217 229 10.1016/S0033-3182(97)71458-0 9136250

[B121] Barker GJ Epstein JB Williams KB Gorsky M Raber-Durlacher JE Current practice and knowledge of oral care for cancer patients: a survey of supportive health care providers Support Care Cancer 2005 13 32 41 10.1007/s00520-004-0691-5 15549427

[B122] Zierler BK Meissner MH Cain K Strandness DE Jr A survey of physicians' knowledge and management of venous thromboembolism Vasc Endovasc Surgery 2002 36 367 375 10.1177/153857440203600506 12244425

[B123] Grimshaw JM Shirran L Thomas R Mowatt GF Bero L Grilli R Harvey E Oxman A O'Brien MA Changing provider behavior: an overview of systematic reviews of interventions Med Care 2001 39 112 145 11583120

[B124] Grimshaw JM Thomas RE MacLennan G Fraser C Ramsay CR Vale L Whitty P Eccles MP Matowe L Shirran L Wensing M Dijkstra R Donaldson C Effectiveness and efficiency of guideline dissemination and implementation strategies Health Technol Assess 2004 8 iii iv 1-72 14960256 10.3310/hta8060

[B125] Jamtvedt G Young JM Kristoffersen DT O'Brien MA Oxman AD Audit and feedback: effects on professional practice and health care outcomes Cochrane Database Syst Rev 2006 2 CD000259 16625533 10.1002/14651858.CD000259.pub2

[B126] Farmer AP Légaré F Turcot L Grimshaw J Harvey E McGowan JL Wolf F Printed educational materials: effects on professional practice and health care outcomes Cochrane Database Syst Rev 2008 3 CD004398 18646106 10.1002/14651858.CD004398.pub2

[B127] Forsetlund L Bjørndal A Rashidian A Jamtvedt G O'Brien MA Wolf F Davis D Odgaard-Jensen J Oxman AD Continuing education meetings and workshops: effects on professional practice and health care outcomes Cochrane Database Syst Rev 2009 2 CD003030 19370580 10.1002/14651858.CD003030.pub2 PMC7138253

[B128] Flodgren G Parmelli E Doumit G Gattellari M O'Brien MA Grimshaw J Eccles MP Local opinion leaders: effects on professional practice and health care outcomes Cochrane Database Syst Rev 2007 1 CD000125 17253445 10.1002/14651858.CD000125.pub3

[B129] Shojania KG Jennings A Mayhew A Ramsay CR Eccles MP Grimshaw J The effects of on-screen, point of care computer reminders on processes and outcomes of care Cochrane Database Syst Rev 2009 3 CD001096 19588323 10.1002/14651858.CD001096.pub2 PMC4171964

[B130] Rx for Change http://www.rxforchange.com

[B131] Health Systems Evidence http://www.healthsystemsevidence.org

[B132] Adams AS Soumerai SB Lomas J Ross-Degnan D Evidence of self-report bias in assessing adherence to guidelines Int J Qual Health Care 1999 11 187 192 10.1093/intqhc/11.3.187 10435838

